# Supplementary material for: VH-replacement shapes the antibody repertoire by removing the genes of non-functional heavy-chains
Source: EMBO J. 2025 Sep 5;44(20):5734–54. doi: 10.1038/s44318-025-00552-8 (PMC12528474; doi:10.1038/s44318-025-00552-8)
Supplement: Supplementary file 1 — Appendix [file 44318_2025_552_MOESM1_ESM.pdf]

Appendix for:

VH-replacement shapes the antibody repertoire by removing the genes of non-functional heavy-chains

Contents:

	Page
Appendix Table S1: VDJseq metadata	1
Appendix Table S2: 89 VH analysed	2
Appendix Table S3: VH-replacement circle	3
Appendix Table S4: VH-replacement donors	14
Appendix Table S5: Primers used for RCseq	15

Appendix Table S1

VDJseq library metadata

Cells	Mouse BM pool	Mice/pool	VDJ reads	DJ reads	VDJ:DJ	proportion alleles VDJ	VDJ alleles/cell	Prod. Seqs	VDJ Productive %
**muMT proB**	1	4	45138	62081	0.73	0.420988817	0.841977635	10480	0.23217688
	2	4	44102	59923	0.74	0.42395578	0.84791156	10185	0.230941907
mean									0.231559394
**muMT proB for RCseq**	3	4							
**ProB**	C	8	28267	47208	0.6	0.374521365	0.749042729	5755	0.203594297
	F	6	37777	56317	0.67	0.401481497	0.802962994	8124	0.215051486
	G	6	36854	53360	0.69	0.408517525	0.81703505	7330	0.198892929
mean									0.205846237
**Large PreB**	A	4	142139	41803	3.4	0.772738146	1.545476291	102352	0.720083862
	B	4	69917	21269	3.29	0.766751475	1.53350295	50135	0.717064519
	C	8	107894	29338	3.68	0.786216043	1.572432086	77423	0.717583925
mean									0.718244102
**Small PreB**	A	4	125989	36802	3.42	0.773930991	1.547861983	91392	0.725396662
	B	4	107739	32224	3.34	0.769767724	1.539535449	77684	0.721038807
	G	6	135015	38950	3.47	0.776104389	1.552208778	96572	0.715268674
mean									0.720568047
**Pro-B subsets**		**Mice/pool**	**VDJ reads**	**DJ reads**	**VDJ:DJ**	**proportion alleles VDJ**	**VDJ alleles/cell**	**Prod. Seqs**	**VDJ Productive %**
	Large cyto-mu neg.	20	12480	76492	0.16315432	0.140268849	0.280537697	708	0.056730769
	Small cyto-mu neg.	20	51873	52119	0.99528003	0.498817217	0.997634433	3794	0.073140169
	Small cyto-mu pos.	20	32740	15549	2.10560165	0.678001201	1.356002402	22615	0.690745266
	Large cyto-mu pos.	20	17241	12904	1.33609733	0.571935644	1.143871289	12388	0.718519807

Appendix Table S2

89 reliably detectable VH

VCALL	Classic name	Clan
IGHV5-2	7183.2.3_(81X)	3
IGHV2-2	Q52.2.4	2
IGHV5-4	7183.4.6	3
IGHV2-3	Q52.3.8	2
IGHV5-6	7183.7.10	3
IGHV2-4	Q52.5.13	2
IGHV5-9	7183.9.15	3
IGHV2-5	Q52.7.18	2
IGHV5-12	7183.12.20	3
IGHV2-6	Q52.8.22	2
IGHV5-9-1	7183.14.25	3
IGHV2-9-1	Q52.9.29	2
IGHV2-6-8	Q52.10.33	2
IGHV5-15	7183.18.35	3
IGHV5-16	7183.19.36	3
IGHV5-17	7183.20.37	3
IGHV2-9	Q52.13.40	2
IGHV7-1	S107.1.42	3
IGHV14-1	SM7.1.44	1
IGHV4-1	X24.1.45	3
IGHV3-1	36-60.1.46	2
IGHV11-1	VH11.1.48	3
IGHV14-2	SM7.2.49	1
IGHV11-2	VH11.2.53	3
IGHV14-3	SM7.3.54	1
IGHV9-1	VGAM3.8-1-57	1
IGHV9-2	VGAM3.8-2-59	1
IGHV9-3	VGAM3.8-3-61	1
IGHV7-3	S107.3.62	3
IGHV14-4	SM7.4.63	1
IGHV3-3	36-60.3.64	2
IGHV3-4	36-60.4.66	2
IGHV3-5	36-60.5.67	2
IGHV3-6	36-60.6.70	2
IGHV9-4	VGAM3.8-4-71	1
IGHV3-8	36-60.8.74	2
IGHV13-2	3609N.2.77	3
IGHV12-3	VH12.1.78	2
IGHV6-3	J606.1.79	3
IGHV6-5	J606.3.81	3
IGHV6-6	J606.4.82	3
IGHV10-1	VH10.1.86	3
IGHV1-4	J558.2.88	1
IGHV1-5	J558.3.90	1
IGHV10-3	VH10.3.91	3
IGHV1-7	J558.4.93	1
IGHV15-2	VH15.1.95	1
IGHV1-9	J558.6.96	1
IGHV1-11	J558.8.98	1
IGHV1-12	J558.9.99	1
IGHV1-15	J558.12.102	1
IGHV1-18	J558.16.106	1
IGHV1-19	J558.18.108	1
IGHV1-22	J558.22.112	1
IGHV1-26	J558.26.116	1
IGHV1-31	J558.31.121	1
IGHV1-34	J558.34.124	1
IGHV1-36	J558.36.126	1
IGHV1-39	J558.39.129	1
IGHV1-42	J558.42.132	1
IGHV1-47	J558.47.137	1
IGHV1-49	J558.49.141	1
IGHV1-50	J558.50.143	1
IGHV1-52	J558.52.145	1
IGHV1-53	J558.53.146	1
IGHV8-6	3609.5.147	2
IGHV1-54	J558.54.148	1
IGHV1-55	J558.55.149	1
IGHV8-8	3609.7.153	2
IGHV1-58	J558.58.154	1
IGHV1-59	J558.59.155	1
IGHV1-61	J558.61.157	1
IGHV1-62-2	J558.64.162	1
IGHV1-63	J558.66.165	1
IGHV1-64	J558.67.166	1
IGHV8-11	3609.11.169	2
IGHV1-66	J558.69.170	1
IGHV1-69	J558.72.173	1
IGHV8-12	3609.12.174	2
IGHV1-72	J558.75.177	1
IGHV1-74	J558.77.180	1
IGHV1-75	J558.78.182	1
IGHV1-76	J558.79.184	1
IGHV1-77	J558.80.186	1
IGHV1-78	J558.81.187	1
IGHV1-80	J558.83.189	1
IGHV1-81	J558.84.190	1
IGHV1-82	J558.85.191	1
IGHV1-85	J558.88.194	1

Appendix Table S3

VH-replacement circle sequences

Key: back to back heptamers +/- insertion, donor/invader nonamer

Sort 2: RCseq after VDJseq, MiSeq Sequencing, shell-based searches for back to back RSS with up to 3 insertions. Pro-B subsets.

LP

>M02293:67:000000000-LFYL9:1:1113:11780:16646 1:N:0:0 GTCCGC reverse complement 5-2 by 1-55 2 insertions
GGGCGATTCACCATCTCCAGAGACAATACCAAGAAGACCCTGTACCTGCAAATGAGCAGT
CTGAGGTCTGAGGACACAGCCTTGTATTACTGTGCCCACAGTGTTGCAACCACATCCTGA
GAGTGTCCAAAACCCTGGAGGAGTAGCAAACTGCCCTGGGACT

SN

>M02293:67:000000000-LFYL9:1:1112:14893:17472 1:N:0:0 ATGTCA reverse complement 5-4 by 2-6-8 (by blast) 0 insertions
GAGAGCGATTCTCATCTCCAGAGACAATGCCAAGAACAACCTGTACCTGCAAATGAGCCA
TCTGAAGTCTGAGGACACAGCCATGTATTACTGTGCACAGTGAGGGAAGTCCAATGTGAG
CCTGCACAAATACTTCTCTGCAGGGATGATCACAACCAGCAGGGGGCGCTGAGGATCCAA
AGGGACT

>M02293:67:000000000-LFYL9:1:1113:22579:13743 1:N:0:0 ATGTCA reverse complement 5-6 by 15-2 0 insertions
GGGCGATTCACCATCTCCAGAGACAATGCCAAGAACACCCTGTACCTGCAAATGAGCAGT
CTGAAGTCTGAGGACACAGCCATGTATTACTGTGCACAGTGTAACAGCTCATATCTGAAG
CATGTCAAAAAGTCTGAAGGCAGGAAGCTATTTTGGGTCTGATATTACCACAAAGATTAA
CCAAAGCAGTTGCTCAAAGCCTGTGGTTAAATGTTGAAGGAAAGA

>M02293:67:000000000-LFYL9:1:1119:25597:9060 1:N:0:0 ATGTCA reverse complement 5-2 by 3-2 Pseudogene 0 insertions
GGGCGATTCACCATCTCCAGAGACAATACCAAGAAGACCCTGTACCTGCAAATGAGCAGT
CTGAGGTCTGAGGACACAGCCTTGTATTACTGTGCACAGTGTGGAGTCTTCACTGTGAGC
CCAGACAAAAACCTCCTTGCAGAGCAGCACTGCACCAACAGGGGGCATGGAGCATACACC
AATAATGGGAAATCTACTTTAAAGTAGGTAAAAAAAAATTGTTGCCCAAAACTCTGGCAC
CAGACTCCAGGTATACAGTAAGAAACATTGGTGCTAATAATTTCAGTCAATATAATGTGC
ACAGCCCAATAAAACTCCTTATGTTATACCCAGAATTCTTCAATGGGAGCATCTTCCAGA
GCTTCCAAATCCCAGCTCAAAACCCAGCTGAAGTCTGACGGCCTCATAGAGGACAAATCA
TT

>M02293:67:000000000-LFYL9:1:2104:3918:15300 1:N:0:0 ATGTCA reverse complement 5-2 by 1-66 0 insertions
GAGAGACGATTCATCATCTCCAGAGACAATACCAAGAAGACCCTGTACCTGCAAATGAGC
AGTCTGAGGTCTGAGGACACAGCCTTGTATTACTGTGCACAGTGTTGTAACCACATCACG
AGTGTGTCAGAAACCCTGGAGAGCAGAAAGCTGCACTGCGACTGAGATGACAGAAGGATT
AATCCTTAGACTTGCTCAGAATTTGTAATTTTGAATGTCCATTCATTACCTCCTCCTCAG
AGTCCTATAGTGCCTTTGTCAGCTTTGTAAATGTCCATCGGTGAGTAAAGTGAAGATATT
TGGATAAACCTAAAATTCTTCACACTTTCTGAATCTATTCACCAGTGACCACCTCC

>M02293:67:000000000-LFYL9:1:1105:5825:8009 1:N:0:0 ATGTCA reverse complement 5-2 by 1- 1 insertion
GGCCGATTCACCATCTCCAGAGACAATACCAAGAAGACCCTGTACCTGCAAATGAGCAGT
CTGAGGTCTGAGGACACAGCCTTGTATTACTGTGCCACAGTGTTGTAACCACATCCTGAG

T

>M02293:67:000000000-LFYL9:1:1118:4543:16029 1:N:0:0 ATGTCA reverse complement 5-2 by 1- 1 insertion
GGCCGATTCACCATCTCCAGAGACAATACCAAGAAGACCCTGTACCTGCAAATGAGCAGT
CTGAGGTCTGAGGACACAGCCTTGTATTACTGTGTCACAGTGCTA

>M02293:67:000000000-LFYL9:1:2111:18651:8400 1:N:0:0 ATGTCA reverse complement 5-4 by 3-1/2(PG) 2 insertions
GGGCGATTCACCATCTCCAGAGACAATGCCAAGAACAACCTGTACCTGCAAATGAGCCAT
CTGAAGTCTGAGGACACAGCCATGTATTACTGTGGTCACAGTGTGGAGTCTTCACTGTGA
GCCCAGACAAAAACCTCCTTGCAGAGCAGCACTGCACCAACAGGGGGCATGGAGCATACA
CCAATAATGGGAAATCTACTTTAAA

>M02293:67:000000000-LFYL9:1:1110:18852:9656 1:N:0:0 ATGTCA reverse complement 5-12 by 2-6*03(by blast) 3 insertions
GGGCGATTCACCATCTCCAGAGACAATGCCAAGAACACCCTGTACCTGCAAATGAGCCGT
CTGAAGTCTGAGGACACAGCCATGTATTACTGTGAAGCACAGTGTGGGAAGTCCAATGTG
AGCCTGCACAAATACTTCTCTGCAGGGATGCTCACAACCAGCAGGGGGCGCTGAGGACCC
AAAGGGACTTCCCAGGATCTCTTCTGGAATCTAGGGAGCTCTGACCTGTGTCTATCAGCA
TGTGTTTCAATGTTAGAGTTCTTAGTTTTCCTTCCAGCAACAGAGATATTTTAGAGCCC

>M02293:67:000000000-LFYL9:1:1119:19064:19452 1:N:0:0 ATGTCA reverse complement 5-6 by 5-9 3 insertions
GGGCGATTCACCATCTCCAGAGACAATGCCAAGAACACCCTGTACCTGCAAATGAGCAGT
CTGAAGTCTGAGGACACAGCCATGTATTACTGTGGGTCACAGTGAGTGAATGTTACTGTG
AGCTCAAACTAAAACCTCCTGAAGAGCACCCAGGACCAGCAGGGGGCTGAGAGAGCACAG
TAACTTG

SP
>M02293:67:000000000-LFYL9:1:1101:21037:3627 1:N:0:0 CCGTCC reverse complement 5-2 by 1-19 0 insertions
GAGAGACGATTCATCATCTCCAGAGACAATACCAAGAAGACCCTGTACCTGCAAATGAGC
AGTCTGAGGTCTGAGGACACAGCCTTGTATTACTGTGCACAGTGCTACAAACACATCCTG
AGTGTGTCAGAAAACTTGGAGGTGCAGCAAGCTCCCTTGGGACTGACAAGGCTTAGAGAA
GGGCCGCTTGCAGATTTGCTT

>M02293:67:000000000-LFYL9:1:1118:16675:6391 1:N:0:0 CCGTCC reverse complement 5-4 by 1-19/36 0 insertions
GGGCGATTCACCATCTCCAGAGACAGTGCCAAGAACAACCTGTACCTGCAAATGAGCCAT
CTGAAGTCTGAGGACACAGCCATGTATTACTGTGCACAGTGCTACAAACACATCCTGAGT
GTGTCAGAAAACTTGGAGGTGCAGCAAGCTCCCTTGGGACTGAC

Sort 3: RCseq, Aviti Sequencing, S. Andrews search; and shell-based searches for back to back RSS with up to 3 insertions. Pro-B pool (DEBC), Pro-B subsets (LN, LP, SN, SP) and large pre-B pool (Cp)

DEBC andrews
>AV240405:AV_A_HW6069_PE300_200624:2409673462:1:10504:1440:0212 reverse complement 5-2 by 1-39 4 insertions
GAGAGACGATTCATCATCTCCAGAGACAATACCAAGAAGACCCTGTACCTGCAAATGAGC
AGTCTGAGGTCTGAGGACACAGCCTTGTATTACTGTGCAGGCACAGTGTTGTAACCACAT
CCTGAGTGTGTCAGAAAACCTGGAGGTGCAGCAAGCTCCCTTAGAACTGACAAGACTTAG

AAAAGTGTTGCTTGTAGATTTGCTTAGATGCAGTCATTTGAATAGTGTGTTTTTGTGTCT
ATTTAGTAAAATCCTATTGTGCTTTTTCAGCTTTGTAGAAGGATATCCATGAATTGCATG
CAGTTTACTAGGATGTCCCTAGAGTACCCCATGCCTTGTCAATATGGATAACAGTGAA

>AV240405:AV_A_HW6069_PE300_200624:2409673462:1:10104:0426:1067
reverse complement 5-2 by 12-3 0 insertions
GAGAGACGATTCATCATCTCCAGAGACAATACCAAGAAGACCCTGTACCTGCAAATGAGC
AGTCTGAGGTCTGAGGACACAGCCTTGTATTACTGTGCACAATGAGAAGATTCCAATGTC
AACCCACACACAAACCTCACTGCAGAGGGGATTTCAACCAGCAGGTGGTGCTGTTCGATG
TAAATGATCAGTAATTTCAAACATCTGTAAAAGAAAAAGAGAATGTCTTTGGCCAGGGCT
AAGTCTTTTCTGTATATGATTATAGGAACATCAGTTGTTCATGAATATCTTTTGGAGAAC
TAAATGACTATAGTGTTTCAATTACCGAG

>AV240405:AV_A_HW6069_PE300_200624:2409673462:1:11003:4897:0161
reverse complement 2-6-8 by 1-26 1 insertion
TCAAATCCAGACTGAGCATCAGCAAGGACAACTCCAAGAGCCAAGTTTTCTTAAAAATGA
ACAGTCTGCAAACTGATGACACAGCCATGTACTACTGTGCCACAGTGCTACAAACACATC
CTGAGTGTGTCAGAAACCCTGGAGGTGCAGCAAGCTCCCTTGGGATTGACAAGACTTAGA
GAATAGCCGCTTGCAGACTTCCTTAGATGCAGTCATTTGAATAGTGTGTTTTTGTGTCTA
TTTCTTAAAGTCCTATTGTGCTTTTTCTGCTTTTCAGAAGAAAATCAATGAATTGCATGC
AGTTTACTAGGATGTCCCTGGAGTACCCCATGCCTTGTCAATATGGATAACAGTGAACGC
TTTCATTGACATTTTTTCTTCTTTCATAAAACCACAC

>AV240405:AV_A_HW6069_PE300_200624:2409673462:1:10204:4020:1534
reverse complement 2-3 by 1-7 0 insertions
TCAAATCCAGACTGAGCATCAGCAAGGATAACTCCAAGAGCCAAGTTTTCTTAAAACTGA
ACAGTCTGCAAACTGATGACACAGCCACGTACTACTGTGCACAGTGGTGCAACCACATCC
CGACTGTGTCAGAAACCCTAGCAGAACAGGAAGCTTCCCTGGGACTGAGAATTCAGAAAA
GACTAACCTGTAGGCTTGATGAAAAATAATCATTTTGGGCACTAATTTTTATGCATGCTC
CTTACACTCTTACAGCGCCTTTTTCAACTATGTAAATA

>AV240405:AV_A_HW6069_PE300_200624:2409673462:1:11301:0028:1956
reverse complement 5-2 by 1-76 0 insertions
GAGAGACGATTCATCATCTCCAGAGACAATACCAAGAAGACCCTGTACCTGCAAATGAGC
AGTCTGAGGTCTGAGGACACAGCCTTGTATTACTGTGCACAGTGTTACAACCACATCCTG
AGTGTGTCAGAAACCCTGTAGGAGCAGGAAGCTGCACTGAGACTGAGATGACAGAAAGAT
TAATCTTTAGACTTGCTCAGAAATTGTAATTTTGAATGTCCATTTATTACCTCCTACTAA
CAGTCGTATAGTATCTTTGTCAACTTTGTCATCGTTTTCTATGAATAAAGTAAGGCTGTT
TGGATAAACTATCAATTCACAATACCTTGTGAATCTCTTCACCATTGA

>AV240405:AV_A_HW6069_PE300_200624:2409673462:1:11202:1313:3316
reverse complement 5-6 by 1-81 0 insertions
GGCCGATTCACCATCTCCAGAGACAATGCCAAGAACACCCTGTACCTGCAAATGAGCAGT
CTGAAGTCTGAGGACACAGCCATGTATTACTGTGCACAGTGTTGTAACCACATCCTGAGT
GTGTCAGAAAACCTGGGGCAGAAGAAAGATAAGCTGGGACTGAGAAGACAGAAAAATTAA
TCCTTAGATTTGCTCAGAAATCATAGTTTTGAATGCCTATTTATTTCCTTCTCCTCACAG
ATCTATAGTGCTTTTGTCAGCTTCTTAAATGTCCATCTATGAGT

>AV240405:AV_A_HW6069_PE300_200624:2409673462:1:10705:0404:2814
reverse complement 2-4 by 1-82 3 insertions
TCATATCCAGACTGAGCATCAGCAAGGACAACTCCAAGAGCCAAGTTTTCTTTAAAATGA
ACAGTCTGCAAGCTGATGACACTGCCATATACTACTATGTCCCACAGTGTTACAACCACA
TCCTGAGAGTGTCAGAAACCGTGGAGGAGCAGGAAGCTTCCCTGGGCCTGAGATGACAGA
AAGATTAATCTTTAGACTTGCTCAGAAATCATAATTACTTTGTAAACATCCATCTATGAA
TAAAGTGATGCTGTTTGGAGAAACCTACACCTTCTGAATCTCTTCACCTGTGACCTGTTT

CTTATTCAATAAAACAATAAAAAGCAAAAGTGTGTTTTTGCATGATAAAAATATTACTGA
ATCCTGAGTGACTAGAACTTTTTTAAAGTTGTTGGGAATAGTCTGTAATAGAAATTTGAT
CTATGTGCAAGCCACCAACATTTGGTCAAACAAACAATGCTTTATAGTCTTATGTAACTT
AGTGACCTCAAGTGTGTTAACTCTTGTCCCATGTTCTAGCAGAAATTCCTGTCTCTTAGA
AGGTG

>AV240405:AV_A_HW6069_PE300_200624:2409673462:1:12005:3725:1678
reverse complement 5-2 by 1-36 0 insertion
GGGCGATTCACCATCTCCAGAGACAATACCAAGAAGACCCTGTACCTGCAAATGAGCAGT
CTGAGGTCTGAGGACACAGCCTTGTATTACTGTGCACAGTGCTACAAACACATCCTGAGT
GTGTCAGAAAACTTGGAGGTGCAGCAAGCTCCCTTGGGACTGACAAGACTTAGAGAAGTG
CCGCTTGCAGATTTGATTAAATGTAATCATTTGAATAGTGTGTTTTTGTGTCTATTTCTT
AAAGACCTATTTTTCTTTTTTAGCTTTGCACAAGGACATCCATGAATTACATGCAGTTTA
CTAGGATGTCCCTGGAGTACCCCCATGCCTTGTCAATATGGATAACAGTGAACACTTTCC
TTGAAAAATTTCTTCTTTCA

DEBC shell

>AV240405:AV_A_HW6069_PE300_200624:2409673462:1:21605:4021:3544
5-2 by 3-6 0 insertions
GAGAGACGATTCATCATCTCCAGAGACAATACCAAGAAGACCCTGTACCTGCAAATGAGC
AGTCTGAGGTCTGAGGACACAGCCTTGTATTACTGTGCACAGTGTGGAGTCTTCACTGTG
AGCCCAGACATAAACCTCCTGTAGAGCAGCTCTGTACCAACAGGGGGCTTGTCACATACA
CTTAGCACAGGAAATCTATTTTAGGGTATGTTAAACACAATTCTTGCCCAAAACTCTGGC
AGGAGACTCCTGCTGAAAAGGGAGACACATTAGTGA

>AV240405:AV_A_HW6069_PE300_200624:2409673462:1:21001:1581:2018
5-4 by 13-1 Pseudogene 0 insertions
GGGCGATTCACCATCTCCAGAGACAATGCCAAGAACAACCTGTACCTGCAAATGAGCCAT
CTGAAGTCTGAGGACACAGCCATGTATTACTGTGCACAGTGTGGAGTCTTCAGTGTAGGC
CCAGACATAAACCTCCTTGTAGAGCAGCTCT

>AV240405:AV_A_HW6069_PE300_200624:2409673462:1:21403:1990:1232
1-11 by 1-84 (not in VH89, low recomb) 0 insertions
AGTTCAAGGGCAAGGCCACATTCTCTGTAGACCGGTCCTCCAGCACAGTGTACATGGTGT
TGAACAGCCTGACATCTGAGGACCCTGCTGTCTATTACTGTGCACAGTGTTACAACCACA
TCCTGAGTGTGTCAGAAACCCTGGATGAGCAGGAAGCTGCACTGCGACTGAGATGACAGA
AAGATTAATCCTTAGACTTGCTCAGAAATTGTAATTTTGAATGTCCATTTATTACCTCCT
CCTCAGAGTCCTATAGTGCCTTTGTCAGCTTTGCAAACGTTCATCTATGAATAATGTCAT
TCTGTTTGGATAAACCTACAGTTCACCATACCTTGTGAATTTCTTCATCCGTGACCACTT
TCCATAAAAAAGAACAAATTTTATTCAATAGAACAAAAAAAGTGAAAAGTATGTTTATAC
ATGATAAAAATATTACTGGATCCTGAGTGATCAG

LP Andrews

>AV240405:AV_A_HW6069_PE300_200624:2409673462:1:20103:2233:0449
reverse complement 2-3 by 1-82 0 insertions
TCAAATCCAGACTGAGCATCAGCAAGGATAACTCCAAGAGCCAAGTTTTCTTAAAACTGA
ACAGTCTGCAAACTGATGACACAGCCACGTACTACTGTGCACAGTGTTACAACCACATCC
TGAGAGTGTCAGAAACCGTGGAGGAGCAGGAAGCTTCCCTGGGCCTGAGATGACAGAAAG
ATTAATCTTTAGACTTGCTCAGAAATCATAATTACTTTGTAAACAT

>AV240405:AV_A_HW6069_PE300_200624:2409673462:1:12102:1974:0559
reverse complement 1-11 by 1-19 0 insertions
CATGGGCAAGGCCACATTCTCTGTAGACCGGTCCTCCAGCACAGTGTACATGGTGTTGAA
CAGCCTGACATCTGAGGACCCTGCTGTCTATTACTGTGCACAGTGCTACAAACACATCCT
GAGTGTGTCAGAAAACTTGGAGGTGCAGCAAGCTCCCTTGGGACTGACAAGGCTTAGAGA

AGGGCCGCTTGCAGATTTGCTTAAATGTAATCATTTGAATACTGTGTTTTTGTGTCTATT
TCTTAAAGACCTATTTTTCTTTTTTAGCTTTGCACAAGGCCATCCATGAATTACATGCAG
TTTACTAGGATGTCCCTGGAGTACCCCCATGCCTTGTCAATATGGATAACAGTGAACACT
TTCCTTGAAAAAATTTCTTCTTTCATAAAACAACAAAAAAATGGAAAATGTGATT

LP shell
>AV240405:AV_A_HW6069_PE300_200624:2409673462:1:10803:0664:0432
5-4 by 1- 3 insertions
GGGCGATTCACCATCTCCAGAGACAATGCCAAGAACAACCTGTACCTGCAAATGAGCCAT
CTGAAGTCTGAGGACACAGCCATGTATTACTGTGGGCCACAGTGTTGTAACC

SN Andrews
>AV240405:AV_A_HW6069_PE300_200624:2409673462:1:11704:2545:2656
reverse complement 5-6 by 9-4 2-insertions
GGGCGATTCACCATCTCCAGAGACAATGCCAAGAACACCCTGTACCTGCAAATGAGCAGT
CTGAAGTCTGAGGACACAGCCATGTATTACTGTGCCCACAGTGTGAAAACCACATCCTGA
GTGTGTCAGAAACCATGAGGAGAAGGTGGTTCAGCTAAGCCCAGACAAAAAGTGAGAAAA
CATTCTCTCCTTCATTATGGACCACAAATACGAGCTTACTGACAATAGATAGAAAATTCA
CATATGGTGAGCCTCAGAATGTTCTCAGTGGGTTGTGACAGACTACCTTAATGAAAGGAG
AAGGGAGTTTAGGGATCAGGTGGGAAGGGGGATGGGGGCATTCA

>AV240405:AV_A_HW6069_PE300_200624:2409673462:1:21402:4565:2313
reverse complement 1-39 by 1-75 2 insertions altered RSS
AGTTCAAGGGCAAGGCCACATTGACTGTAGACCAATCTTCCAGCACAGCCTACATGCAGC
TCAACAGCCTGACATCTGAGGACTCTGCAGTCTATTACTGTGCCTTCAGTGTTACAACCA
CATCCTGAGTGTGTCAGAAACCCTGGAGGAGCAGGAAGCTGCACTGGGACTGAGATGACA
GAAAGATTAATCCTTAGACTTTCTCAGAATTTTAATTCTGAATGTCCATTTATTACCTCC
TCCTCAGAGTCCTATAGTGCCTTTGTCAGCTTTGTAAATGTCCATCTATGAGTAAAGTGA
T

>AV240405:AV_A_HW6069_PE300_200624:2409673462:1:10104:3147:0858
reverse complement 1-11 by 8-12 4 insertions altered RSS
CATGGGCAAGGCCACATTCTCTGTAGACCGGTCCTCCAGCACAGTGTACATGGTGTTGAA
CAGCCTGACATCTGAGGACCCTGCTGTCTATTACTGTGGAATAATTTTGATACAGCTTAA
GTTTTCAGCTGTACAGTATTTCAGGCTGGTGTTCAGCTCTGTTCCTTCCAATTATATCAT
AAGAGGGTATGGCCGCAGTTTTCCTCTTGCCTCTGTGGTTTCCTGTTTGCTCTTAGGTTA
AGAGCTTTCTCTTCCTGTTATTCATCC

>AV240405:AV_A_HW6069_PE300_200624:2409673462:1:10104:2610:3463
reverse complement 5-2 by 1-77 0 insertions
GAGAGACGATTCATCATCTCCAGAGACAATACCAAGAAGACCCTGTACCTGCAAATGAGC
AGTCTGAGGTCTGAGGACACAGCCTTGTATTACTGTGCACAGTGTTGTAACCACATCCTG
AGTGTGTCAGAAACACTGGAGGAGCAGGAAGCTGCACTGGGACTGAGATGACAGAAAGAT
TAATCCTTAGACTTGCTCAGAAATTGTAATTTTGAATGTCCATGTATTG

>AV240405:AV_A_HW6069_PE300_200624:2409673462:1:12004:0643:2327
reverse complements 5-4 by 1-26 0 insertions
GGGCGATTCACCATCTCCAGAGACAATGCCAAGAACAACCTGTACCTGCAAATGAGCCAT
CTGAAGTCTGAGGACACAGCCATGTATTACTGTGCACAGTGCTACAAACACATCCTGAGT
GTGTCAGAAACCCTGGAGGTGCAGCAAGCTCCCTTGGGATTGACAAGACTTAGAGAATAG
CCGCTTGCAGACTTCCTTAGATGCAGTCATTTGAATAGTGTGTTTTTGTGTCTATTTCTT
AAAGTCCTATTGTGCTTTTTCTGCTTTTCAGAAGAAAATCAATGAATTGCATGCAGTTTA
CTAGGATGTCCCTGGAGTACCCCATGCCTTGTCAATATGGATAACAGTGAACGCTTTCAT

TGACATTTTTTCTTCT

>AV240405:AV_A_HW6069_PE300_200624:2409673462:1:12004:4401:3484
reverse complement 5-2 by 1-82 0 insertions
GAGAGACGATTCATCATCTCCAGAGACAATACCAAGAAGACCCTGTACCTGCAAATGAGC
AGTCTGAGGTCTGAGGACACAGCCTTGTATTACTGTGCACAGTGTTACAACCACATCCTG
AGAGTGTCAGAAACCGTGGAGGAGCAGGAAGCTTCCCTGGGCCTGAGATGACAGAAAGAT
TAATCTTTAGACTTGCTCAGAAATCATAATTACTTTGTAAACATCCATCTATGAATAAAG
TGATGCTGTTTGGAGAAACCTACACCTTCTGAATCTCTTCACCTGTGACCTGTTTCTTAT
TCAATAAAACAATAAAAAGCAAAAGTGTGTTTTTGCATGATAAAAAAATTACTGAATCCT
GAGTGACTAGAACTTTTTTAAAGTTGTTGGGAATAGTCTGTAATAGAAATTTGATCTATG
TGCAAGCCACCAACATTTGGTCAAACAAACAATGCTTTATAGTCTTATGTA

>AV240405:AV_A_HW6069_PE300_200624:2409673462:1:10203:5301:2116
reverse complement 3-1 by 1-69 4 insertions all but 1 of 3-1 3’
CCCTCAAAAGTCGAATCTCCATCACTCATGACACATCTAAGAACCATTTCTTCCTGAAGT
TGAATTCTGTGACTACTGAGGACACAGCCACATATTACTGTGCAAGAGGAGTGTTGCAAC
CACATCCTGAGAGTGTCAGAAACCCTGGAGGAGTAGCAAACTGCCCTGAGACTGAGGAGA
CTCAGAGAAGGTTTGCTTGTAGACTTGCTCAGATACAGCCAGGATGGTGTGTAGTATGGG
CCCCAGACATGTA

>AV240405:AV_A_HW6069_PE300_200624:2409673462:1:20401:3658:2741
reverse complement 2-2 by 1-7 0 insertions
TCATATCCAGACTGAGCATCAGCAAGGACAATTCCAAGAGCCAAGTTTTCTTTAAAATGA
ACAGTCTGCAAGCTGATGACACAGCCATATATTACTGTGCACAGTGGTGCAACCACATCC
CGACTGTGTCAGAAACCCTAGCAGAACAGGAAGCTTCCCTGGGACTGAGAATTCAGAAAA
GACTAACCTGTAG

>AV240405:AV_A_HW6069_PE300_200624:2409673462:1:22101:4882:1934
reverse complement 2-4 by 5-12 0 insertions
TCATATCCAGACTGAGCATCAGCAAGGACAACTCCAAGAGCCAAGTTTTCTTTAAAATGA
ACAGTCTGCAAGCTGATGACACTGCCATATACTACTGTGCACAATGAGGAAATGTTACTG
TGAGCTCAAACTAAAACCTCCTGCAGAGCACCCAGGACCAGCAGGGGGCGCAGAGAGCAC
ATGGAGTTCTGATTCACAGAAGAGTTACAGCCTGTACAATTAGACCCAATCTTCAACAAA
CCGTCAAAATATTCGATCCAAAATTGTTCCTGTCTAAAAGTAATTCAAGGACAAAATGGA
CCAGAGACTGAAGAAATGGCTGACCTGTGACCCTCCCAACTTTGGATCTATCTCATAGGC
AGGTACCAAACCTTGACATTTGTCACTGACACTGTATTGTGCTTGCAGACAGGAGCATAG
CATGGCTGACCTCTAAGAGGCTCTGCAAGCACCTGAATGAGACAGATG

SP andrews
>AV240405:AV_A_HW6069_PE300_200624:2409673462:1:20102:0907:2368
reverse complement 5-2 by 1-55 0 insertions
GAGAGACGATTCATCATCTCCAGAGACAATACCAAGAAGACCCTGTACCTGCAAATGAGC
AGTCTGAGGTCTGAGGACACAGCCTTGTATTACTGTGCACAGTGTTGCAACCACATCCTG
AGAGTGTCCAAAACCCTGGAGGAGTAGCAAACTGCCCTGGGACTGTGGAGACTCAGAGAA
GTTTTGCTTGTAGACTTGCTCAGATACAGCCATTTAGATAGTCCAATTTTGTGTTTGTTA
CTTAGAGTTCTGTAGTGCTTTTGTCCACTTTGCAAATGGACATCGATTAATCATATGATA
TTGATTTAGGATGCTTTGAGAGTATGCCATACCTTGTCAATTACCTTCTTCACCAGTGAC
CATCTCTGTTGACATATTTCTTTTGTA

>AV240405:AV_A_HW6069_PE300_200624:2409673462:1:10103:3941:0457
reverse complement 3-1 by 15-2 0 insertions

CCCTCAAAAGTCGAATCTCCATCACTCATGACACATCTAAGAACCATTTCTTCCTGAAGT
TGAATTCTGTGACTACTGAGGACACAGCCACATATTACTGTGCACAGTGTAACAGCTCAT
ATCTGAAGCATGTCAAAAAGTCTGAAGGCAGGAAGCTATTTTGGGTCTGATATTACCACA
AAGATTAACCAAAGCAGTTGCTCAAAGCCTGTGGTTAAATGTTGAA

>AV240405:AV_A_HW6069_PE300_200624:2409673462:1:10104:4315:0576
reverse complement 5-2 by 1-9 0 insertions
GAGAGACGATTCATCATCTCCAGAGACAATACCAAGAAGACCCTGTACCTGCAAATGAGC
AGTCTGAGGTCTGAGGACACAGCCTTGTATTACTGTGCACAGTGTTGTAACCACATCCTG
AGTGTGTCAGAAACTCTGGAGGAGCAGCAAGCTGCCCTGGGTCTGAAATATCAGAAAAGG
CTAACATTTAGACTTCCTCAGAAATAACCATTTTGAGTGCCTATTTTTCGTTTGTTAGTT
TCCTACACACAGACTTATAGTGCCTTTGTCAAC

>AV240405:AV_A_HW6069_PE300_200624:2409673462:1:20104:0057:3384
reverse complement 5-2 by 12-3 0 insertions
GAGAGACGATTCATCATCTCCAGAGACAATACCAAGAAGACCCTGTACCTGCAAATGAGC
AGTCTGAGGTCTGAGGACACAGCCTTGTATTACTGTGCACAATGAGAAGATTCCAATGTC
AACCCACACACAAACCTCACTGCAGAGGGGATTTCAACCAGCAGGTGGTGCTGTTCGATG
TAAATGATCAGTAATTTCAAACATCTGTAAAAGAAAAAGAGAATGTCTTTGGCCAGGGCT
AAGTCTTTTCTGTATATGATTATAGGAACATCAGTTG

>AV240405:AV_A_HW6069_PE300_200624:2409673462:1:20601:2543:1099
reverse complement 2-3 by 1-9 0 insertions
TCAAATCCAGACTGAGCATCAGCAAGGATAACTCCAAGAGCCAAGTTTTCTTAAAACTGA
ACAGTCTGCAAACTGATGACACAGCCACGTACTACTGTGCACAGTGTTGTAACCACATCC
TGAGTGTGTCAGAAACTCTGGAGGAGCAGCAAGCTGCCCTGGGTCTGAAATATCAGAAAA
GGCTAACATTTAGACTTCCTCAGAAATAACCATTTTGAGTGCCTATTTTTCGTTTGTTAG
TTTCCTACACACAGACTTATAGTGCCTTTGTCAACTTTGCATAAG

>AV240405:AV_A_HW6069_PE300_200624:2409673462:1:10301:3991:1183
reverse complement 5-2 by 6-3 3 insertions
GAGAGACGATTCATCATCTCCAGAGACAATACCAAGAAGACCCTGTACCTGCAAATGAGC
AGTCTGAGGTCTGAGGACACAGCCTTGTATTACTGTGAGACACAGTGAGAAGTCTTCATT
GTGAGTCTAGACACAAACTTACCCAAAGGAGCTCTCAGTACCAGCAGGGGGAGCACAGTG
ACAATCGAATCCATAAATGGGCTATTGTTTACAGGGATCTGGGCAGGTGAGACCACTTTC
TCAGTAGTCCCTTTCCTTCCTCCACCATCTGGAG

SP shell
>AV240405:AV_A_HW6069_PE300_200624:2409673462:1:21405:0623:2734
5-2 by 8-7 Pseudogene 0 insertions
GAGAGACGATTCATCATCTCCAGAGACAATACCAAGAAGACCCTGTACCTGCAAATGAGC
AGTCTGAGGTCTGAGGACACAGCCTTGTATTACTGTGCACATTGACACAGCCTCAGTTTT
CATCTGTACATTATTTCAGGCTGATTCTCAGTTGTGTTTCTTTCTTTTATATTACAAGAG
GGTATGGCCACAGATTTCCTCTGACCTCTGTGGTTTCCTGTTTGCTCTTAGGTCAAGAGC
CCTCTTCCTGTTATTCACTGTAGGTCCCTATTAATGTGTTCATAATTGATTCTGTCTGTG
TTTTGCTTTTAAAGCCGATGGAGGTTTGGTGCCTACACCAGGTGGGTCAGAGTTATAGTT
TCACTCAGTTTTAGACTTCAAATTCCCACAGTAGAATACTCCAAATTATTGTAATTTTCA
ACTTCAGTAAACAGCTGAAGATGGGAATTACTGGATGTGTATTCTCAAATTGAGTCATTG
AAAAAAAGAGTGTTC

Cp Andrews
>AV240405:AV_A_HW6069_PE300_200624:2409673462:1:11503:0524:0529
reverse complement 5-4 by 9-2 2 insertions
GGCCGATTCACCATCTCCAGAGACAATGCCAAGAACAACCTGTACCTGCAAATGAGCCAT
CTGAAGTCTGAGGACACAGCCATGTATTACTGTGCGCACAGTGTGAAAACCACATCCTGA

GGGTGTCAGAAACCATGAGGAGAAGGTGGTTCAGCTGTGTCCAGAAGCAACCAGAGGAAA
CATTCTCTCCTTGATGTTTGGCCACAATTATGAGATTACTGACAACACATATAATAGTCA
TATATGGTCAGCCACAGAATGTTCTCAGTGGGATTGTGACAGATCAGATTGATGAAAGGA
AGAGGGACCGTTTCATAGCATCTTTAATTTGGTATATATAAACTGTTAATATGAAAATAT
CA

>AV240405:AV_A_HW6069_PE300_200624:2409673462:1:21403:1064:3470
reverse complement 2-2 by 1-26 0 insertions
TCATATCCAGACTGAGCATCAGCAAGGACAATTCCAAGAGCCAAGTTTTCTTTAAAATGA
ACAGTCTGCAAGCTGATGACACAGCCATATATTACTGTGCACAGTGCTACAAACACATCC
TGAGTGTGTCAGAAACCCTGGAGGTGCAGCAAGCTCCCTTGGGATTGACAAGACTTAGAG
AATAGCCGCTTGCAGACTTCCTTAGATGCAGTCATTTGAATAGTGTGTTTTTGTGTCTAT
TTCTTAAAGTCCTATTGTGCTTTTTCTGCTTTTCAGAAGAAAATCAATGAATTGCATGCA
GTTTACTAGGATGTCCCTGGAGTACCCCATGCCTTGTCAATATGGATAACAGTGAACGCT
TTCATTGACATTTTTTCTTCTTTCATAAAACCACACAAAAGGGAAAATGTGATTATGCAT
GATAAAAGTATGTTTTTATTGTGTAAAAGACTTGAACTTTGGAATAGGTGGGAATGAATT
ATCATAGGAATTTGACCCGTAAAATTC

>AV240405:AV_A_HW6069_PE300_200624:2409673462:1:10201:3934:2129
reverse complement 2-2 by 1-19 0 insertions
TCAAATCCAGACTGAGCATCAGCAAGGACAATTCCAAGAGCCAAGTTTTCTTTAAAATGA
ACAGTCTGCAAGCTGATGACACAGCCATATATTACTGTGCACAGTGCTACAAACACATCC
TGAGTGTGTCAGAAAACTTGGAGGTGCAGCAAGCTCCCTTGGGACTGACAAGGCTTAGAG
AAGGGCCGCTTGCAGATTTGCTTAAATGTA

>AV240405:AV_A_HW6069_PE300_200624:2409673462:1:10703:5212:2446
reverse complement 2-2 by 1-22 0 insertions
TCAAATCCAGACTGAGCATCAGCAAGGACAATTCCAAGAGCCAAGTTTTCTTTAAAATGA
ACAGTCTGCAAGCTGATGACACAGCCATATATTACTGTGCACAGTGCTACAAACACATCC
TCAGTGTGTCAGAAATCCTGGAGGTGCAGCAAGCTCCCTTGGGACTGACAAGACTTAGAG
AATAGTTGCTTGCAGACGTGCTTAGGTGCAGACATTTGGATAGTGTGTTTTTGTGTCTAT
TTCTTAAAGACCTAGTATGCTTTTTCAGCTTTTCAGAAGAAAATCAAT

>AV240405:AV_A_HW6069_PE300_200624:2409673462:1:20802:4607:3201
reverse complement 5-2 by 1-39 0 insertions
GAGAGACGATTCATCATCTCCAGAGACAATACCAAGAAGACCCTGTACCTGCAAATGAGC
AGTCTGAGGTCTGAGGACACAGCCTTGTATTACTGTGCACAGTGTTGTAACCACATCCTG
AGTGTGTCAGAAAACCTGGAGGTGCAGCAAGCTCCCTTAGAACTGACAAGACTTAGAAAA
GTGTTGCTTGTAGATTTGCTTAGATGCAGTCATTTGAATAGTGTGTTTTTGTGTCTATTT
AGTAAAATCCTATTGTGCTTTTTCAGCTTTGTAGAAGGATATCCATGAATTGCATGCAGT
TTACTAGGATGTCCCTAGAGTACCCCATGCCTTGTCAATATGGATAACAGTGAACACTTT
CATTGACTTATTTCTTCTT

>AV240405:AV_A_HW6069_PE300_200624:2409673462:1:10404:2516:0548
reverse complement 5-2 by 14-1 0 insertions
GAGAGACGATTCATCATCTCCAGAGACAATACCAAGAAGACCCTGTACCTGCAAATGAGC
AGTCTGAGGTCTGAGGACACAGCCTTGTATTACTGTGCACAGTCTTGCAAACACATCCTG
AGAGTGTCATAAACCATAAAGTGCAGGAAGCTGCCTGGAACTGAGATGACAGACTATACT
ACCCTGAAGAAATAGCCATTTTGAGTGTCCCAATTTCTGCCTCCTCCTCA

>AV240405:AV_A_HW6069_PE300_200624:2409673462:1:20401:1492:3626
reverse complement 2-2 by 9-3 4 insertions
TCATATCCAGACTGAGCATCAGCAAGGACAATTCCAAGAGCCAAGTTTTCTTTAAAATGA
ACAGTCTGCAAGCTGATGACACAGCCATATATTACTGTGATCTCACAGTGTGAAAACCAC
ATCCTGAGGGTGTCAAAAACCATGAGGAGAAGGTGGTTCAGCTGTGTCCAGAAGCAACCA
GAGGAAACATTCTCTCCTTGGTGTTTGGCCACAATTATGAGATTACTGACAACACATATA

ATAGTCA

>AV240405:AV_A_HW6069_PE300_200624:2409673462:1:10604:0989:1981 reverse complement 5-2 by 1-19 3 insertions
GAGAGACGATTCATCATCTCCAGAGACAATACCAAGAAGACCCTGTACCTGCAAATGAGC
AGTCTGAGGTCTGAGGACACAGCCTTGTATTACTGTGGCCCACAGTGCTACAAACACATC
CTGAGTGTGTCAGAAAACTTGGAGGTGCAGCAAGCTCCCTTGGGACTGACAAGGCTTAGA
GAAGGGCCGCTTGCAGATTTGCTTAAATGTAATCATTTGAATACTGTGTTTTTGTGTCTA
TTTCTTAAAGACCTATTTTTCTTTT

Cp shell
>AV240405:AV_A_HW6069_PE300_200624:2409673462:1:12104:0250:1332
5-2 by 8-2 (ORF) 0 insertions
GAGAGACGATTCATCATCTCCAGAGACAATACCAAGAAGACCCTGTACCTGCAAATGAGC
AGTCTGAGGTCTGAGGACACAGCCTTGTATTACTGTGCACAGTGGTGCAACCGTGACCCA
CAGCTGTGCAATATTTCAGGATGGATCTCATTTCTGTTCCTTCCTAATAGAGAGGAGGAG
TTGGAGCGGGTCCACATTTCCTGCTAGCCTCTGGGGTTTCCTTTTTACTGTGAGCTTCAG
AGGCTAGGCTTCCTGTTACGCCCCCTAAGTCATTTTTCAAACCTTTTTGTTGTTTCTTTA
TAGATTTGACATCATGAACCCCATTTTTACTCATCTTCCCCTCTCCCACACACCCACTCT
CCAACCTTGGAACCTCCTCCCCAA

>AV240405:AV_A_HW6069_PE300_200624:2409673462:1:20203:3292:1480
5-12 by 2-6*03 0 insertions
GGCCGATTCACCATCTCCAGAGACAATGCCAAGAACACCCTGTACATGCAAATGAGCCGT
CTGAAGTCTGAGGACACAGCCATGTATTACTGTGCACAGTGTGGGAAGTCCAATGTGAGC
CTGCACAAATACT

>AV240405:AV_A_HW6069_PE300_200624:2409673462:1:10603:4885:0698
5-2 by 1-18 to 36, 0 insertions
GAGAGACGATTCATCATCTCCAGAGACAATACCAAGAAGACCCTGTACCTGCAAATGAGC
AGTCTGAGGTCTGAGGACACAGCCTTGTATTACTGTGCACAGTGCTACAAACA

WT vs muMT RCseq sequences. Shell script search

WT sequences

5-2 by 2-6-8
>AV240405:AV_A_HW6219_PE300_02052025:2438512377:1:10102:3802:0656
GAGAGACGATTCATCATCTCCAGAGACAATACCAAGAAGACCCTGTACCTGCAAATGAGCAGTCTGAG
GTCTGAGGACACAGCCTTGTATTACTGTGCACAGTGAGGGAAGTCCAATGTGAGCCTGCACAAATACT
TCTCTGCAGGGATGATCACAACCAGCAGGGGGCGCTGAGGATCCAAAGGGACTTCCCAGGATCTCTTC
TGGAATCTAGGGAGCTCTGGCCTGTGTCTATCAGCATGTGTTTCAATGTTAGAGTTCTGAGTTTTCCT
TCCAGCCACAGAGATTCTTTAGAGCCCACTTTTCATTGTCATTCTACTAAATGTGTTCACACAGTGGA
AGGATTTGTTAAA
--
5-2 by 1-2/5
>AV240405:AV_A_HW6219_PE300_02052025:2438512377:1:10102:5441:2744
GAGAGACGATTCATCATCTCCAGAGACAATACCAAGAAGACCCTGTACCTGCAAATGAGCAGTCTGAG
GTCTGAGGACACAGCCTTGTATTACTGTGCACAGTGTTGTGACCACATCCTGAGTATGTCAGAAAACT
TGGAGGTGTAGGAAGCTGCCCTAGGACTGAGAGTACAGAAAAGATTAACCTGTAGACTTGATCAAAAA
TAATCATTTTGGGCATCCATTTTTCTGTATACTCCTTACACTCTTATAGTGACTTTTTCAACTATGTA
ATTTGATATGTAAAAATAAAGTGGTTCTGATTTAGGATTAACCTTCCTTTCACCATGCCTTGTCACTC
TTGACTACAACCATAAAAATGTTTCTTTTTCAGCAAAACAGTAAGAAAGGGAAAACCAGGA
--

```
2-6 by 12-3
>AV240405:AV_A_HW6219_PE300_02052025:2438512377:1:20103:5006:1807
TCATATCCAGACTGAGCATCAGCAAGGACAACTCCAAGAGCCAAGTTTTCTTAAAAATGAACAGTCTG
CAAACTGATGACACAGCCATGTACTACTGTGCACAATGAGAAGATTCCAATGTCAACCCACACACAAA
CCTCACTGCAGAGGGGATTTCAACCAGCAGGTGGTGCTGTTCGATGTAAATGATCAGTAATTTCAAAC
ATCTGTAAAAGAAAAAGAGAATGTCTTTGGCCAGGGCTAAGTCTTTTCTGTATATGATTATAGGAACA
TCAGTTGTTCATGAATATCTTTTGGAGAACTAAATGACTATAGTGTTTCAATTACCGAGAGCAAGATA
AACACAGGTCAGCAAAAAGCACAGTGTGGAGGTGAAAACCAAAGGATCCTTTGTCTGTCACCTAACAC
GAGTGTGAGGATCAGTCCACAT
--
5-2 by 1-26
>AV240405:AV_A_HW6219_PE300_02052025:2438512377:1:10203:4591:2455
GAGAGACGATTCATCATCTCCAGAGACAATACCAAGAAGACCCTGTACCTGCAAATGAGCAGTCTGAG
GTCTGAGGACACAGCCTTGTATTACTGTGCACAGTGCTACAAACACATCCTGAGTGTGTCAGAAACCC
TGGAGGTGCAGCAAGCTCCCTTGGGATTGACAAGACTTAGAGAATAGCCGCTTGCAGACTTCCTTAGA
TGCAGTCATTTGAATAGTGTGTTTTTGTGTCTATTTCTTAAAGTCCTATTGTGCTTTTTCTGCTTTTC
AGAAGAAAATCAATGAATTGCATGCAGTTTACTAGGATGTCCCTGGAGTACCCCATGCCTTGTCAATA
TGGATAACAGTGAACGCTTTCATTGACATTTTTTCTTCTTTCATAAAACCACACAAAAGGGAAAATGT
GATTATGCATGATAAAAGTATGTTTTTATTGTGTAAAAGACTTGAACTTTGGAATAGGTGGGAATGAA
TTATCATAGGAATTTG
--
5-2 by 10-3
>AV240405:AV_A_HW6219_PE300_02052025:2438512377:1:20201:4986:2946
GGGCGATTCACCATCTCCAGAGACAATACCAAGAAGACCCTGTACCTGCAAATGAGCAGTCTGAGGTC
TGAGGACACAGCCTTGTATTACTGTGGGGCACAGTGTGGAATCTTCAATGTGAGCCTAGACACAAACC
TCCAGTTCAGGTACTCATTACCAGCAGGGGGTGCTTAGTACACACAGAGACAAGATCAGCTCAGAT
```

MuMT1 sequences

```
5-9-1 by 1-59
>AV240405:AV_A_HW6219_PE300_02052025:2438512377:1:20704:3262:2459
GGGCGATTCACCATCTCCAGAGACAATGCCAAGAACACCCTGTACCTGCAAATGAGCAGTCTGAGGTC
TGAGGACACGGCCATGTATTACTGTGCACAGTGTTGCAACCACATCCTGAGAGTGTCAGAAACCCTGG
AGGAGCAGCAAGCTGCCCTGGGACTGAG
--
2-2 by 9-4
>AV240405:AV_A_HW6219_PE300_02052025:2438512377:1:20602:1329:3395
TCATATCCAGACTGAGCATCAGCAAGGACAATTCCAAGAGCCAAGTTTTCTTTAAAATGAACAGTCTG
CAAGCTAATGACACAGCCATATATTACTGTGGGCCACAGTGTGAAAACCACATCCTGAGGGTGTCAGA
AACCATGAGGGAAGGTG
```

MuMT3 sequences

```
5-4 by 8-2
>AV240405:AV_A_HW6219_PE300_02052025:2438512377:1:10102:5293:0041
GGGCGATTCACCATCTCCAGAGACAATGCCAAGAACAACCTGTACCTGCAAATGAGCAGTCTGAAGTC
TGAGGACACAGCCATGTATTACTGTGCACAGTGGTGCAACCGTGACCCACAGCTGTGCAATATTTCAG
GATGGATCTCATTTCTGTTCCTTTCTAATAGAGAGGAGGAGTTGGAGAGGGTACACATTTCCTGCTAG
CCTCTGGGGTTTCCTTTTTACTGTGAGCTTCAGAGGCTAGGCTTCCTGTTATGTGCCCTAAGTCATTT
TTCAAACCTTTTTGTTGTTTCTTTATAGATTTGACATCATGAACCCCAATTTTACTCATCTTCCCCTC
TCCCACACACC
--
5-9-1 by 3-1
>AV240405:AV_A_HW6219_PE300_02052025:2438512377:1:20104:1199:2239
GGGCGATTCACCATCTCCAGAGACAATGCCAAGAACACCCTGTACCTGCAAATGAGCAGTCTGAGGTC
TGAGGACACGGCCATGTATTACTGTGCACAGTGTGAGGTCTTTAGTGTGAGCCCAGACATAAACCTCC
```

TTGCAGAGCAGCACTGCACCAACAGGGGGCGTGGAGCATACACCAATAATGGGAAATCTACTCTAAAG
TAGGTAAAAAAAAAATTGTTGCCC

Appendix Table S4

VH-replacement donors and recipients

Sample	Recipient	Donor	Insertions
LP2	5-2	1-55	2
SN2	5-4	2-6-8	0
SN2	5-6	15-2	0
SN2	5-2	3-2 PG	0
SN2	5-2	1-66	0
SN2	5-2	1-x	1
SN2	5-2	1-x	1
SN2	5-4	3-1/2PG	2
SN2	5-12	2-6	3
SN2	5-6	5-9	3
SP2	5-2	1-19	0
SP2	5-4	1-19/36	0
DEBC	5-2	1-39	4
DEBC	5-2	12-3	0
DEBC	2-6-8	1-26	1
DEBC	2-3	1-7	0
DEBC	5-2	1-76	0
DEBC	5-6	1-81	0
DEBC	2-4	1-82	3
DEBC	5-2	1-36	0
DEBC	5-2	3-6	0
DEBC	5-4	13-1 PG	0
DEBC	1-11	1-84	0
LP3	2-3	1-82	0
LP3	1-11	1-19	0
LP3	5-4	1-x	3
SN3	5-6	9-4	2
SN3	1-39	1-75	2
SN3	1-11	8-12	4
SN3	5-2	1-77	0
SN3	5-4	1-26	0
SN3	5-2	1-82	0
SN3	3-1	1-69	4
SN3	2-2	1-7	0
SN3	2-4	5-12	0
SP3	5-2	1-55	0
SP3	3-1	15-2	0
SP3	5-2	1-9	0
SP3	5-2	12-3	0
SP3	2-3	1-9	0
SP3	5-2	6-3	3
SP3	5-2	8-7 PG	0
Cp	5-4	9-2	2
Cp	2-2	1-26	0
Cp	2-2	1-19	0
Cp	2-2	1-22	0
Cp	5-2	1-39	0
Cp	5-2	14-1	0
Cp	2-2	9-3	4
Cp	5-2	1-19	3
Cp	5-2	8-2 ORF	0
Cp	5-12	2-6	0
Cp	5-2	1-18to36	0

Appendix Table S5

primers for VH replacement circle sequencing

Name	Sequence
VH1-11bio	CCAGTAAGTGGTGAAACTAACTACAATC
VH1-11P5	GTGACTGGAGTTCAGACGTGTGCTCTTCCGATCTCATGGGCAAGGCCACATTCTCT*G
VH2-2bio	GGAGTGGTGGAAGCACAGAC
VH2-3bio	GGGGTGACGGGAGCACAAAT
VH2-61bio	TAATATGGGGTGTTGGAAGCACAAAT
VH2-62bio	ATATGGAGTGATGGAAGCACAACC
VH2-2/3/6	GTGACTGGAGTTCAGACGTGTGCTCTTCCGATCTTCAWATCCAGACTGAGCATCAGCAA*G
VH5-2bio	GATGGTGGTAGCACCTACTATCC
VH5-4/6bi	TGGTAGTTACACCTACTATCCAGAC
VH5-12bio	GTGGTAGCACCTATTATCCAGAC
VH5-2P5	GTGACTGGAGTTCAGACGTGTGCTCTTCCGATCTGAGAGACGATTCATCATCTCCAGA*G
VH5-4/6/1	GTGACTGGAGTTCAGACGTGTGCTCTTCCGATCTGGSCGATTCACCATCTCCAGA*G
VH3-1/6bi	TGGTAGCAMTAACTACAACCCATC
VH3-1/6P5	GTGACTGGAGTTCAGACGTGTGCTCTTCCGATCTCYCTCAAAARTCGAATCTCCATCACT*C
VH1-26bio	ATTAATCCTAACAATGGTGGTACTAGC
VH1-81bio	CCTAGAAGTGGTAATACTTACTACAATG
VH1-26/81	GTGACTGGAGTTCAGACGTGTGCTCTTCCGATCTAGTTCAAGGGCAAGGCCACAYT*G
