# Supplementary material for: VH-replacement shapes the antibody repertoire by removing the genes of non-functional heavy-chains
Source: EMBO J. 2025 Sep 5;44(20):5734–54. doi: 10.1038/s44318-025-00552-8 (PMC12528474; doi:10.1038/s44318-025-00552-8)
Supplement: Supplementary file 9 — Expanded View Figures [file 44318_2025_552_MOESM9_ESM.pdf]

Expanded View Figures

Figure EV1. FACS gating strategy and VDJseq library replicate comparisons.

(**A**) Babraham Institute FACS gating strategy for selection of pro-B cells, large pre-B cells and small pre-B cells. (**B**) Diagram showing that some c-kit+/CD43+/IgM- bone marrow B-cells overlap with CD25-/IL-7R+/IgM-/CD24+/CD43lo gate for large pre-B cells. (**C**) Frequency of individual VH in VDJseq libraries from biological replicate libraries from mouse bone marrow pools. Consult Appendix Table S1 for composition of bone marrow B-cell pools. (**D**) VH frequency in wild-type pro-B non-productive VDJ versus µMT pro-B non-productive VDJ. Data Information: Data derived from RStudio analysis of merged biological replicate datasets of VDJseq analysis of respective cell types, as Fig. 1. R^2 values calculated using simple linear regression. Source data are available online for this figure.

© The Author(s)

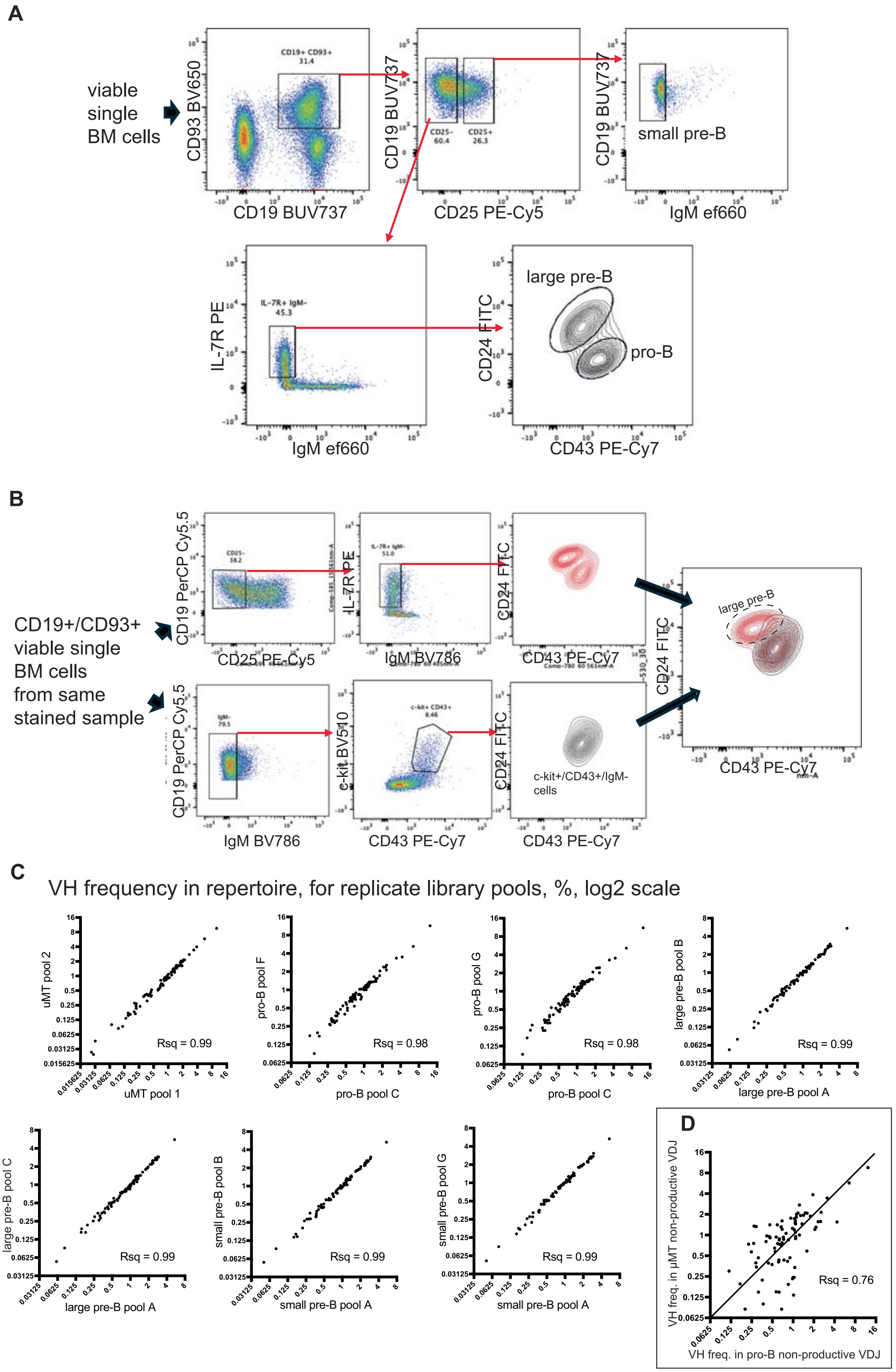
A
viable single BM cells
CD93 BV650
CD19 BUV737
CD19+ CD93+ 31.4
CD25 PE-Cy5
CD25- 60.4
CD25+ 26.3
IgM ef660
small pre-B
IL-7R PE
IL-7R+ IgM- 45.3
CD24 FITC
CD43 PE-Cy7
large pre-B
pro-B
B
CD19+/CD93+ viable single BM cells from same stained sample
CD19 PerCP Cy5.5
CD25 PE-Cy5
IgM BV786
c-kit BV510
c-kit+/CD43+/IgM- cells
C
VH frequency in repertoire, for replicate library pools, %, log2 scale
uMT pool 2
uMT pool 1
Rsq = 0.99
pro-B pool F
pro-B pool C
Rsq = 0.98
pro-B pool G
large pre-B pool B
large pre-B pool A
large pre-B pool C
small pre-B pool B
small pre-B pool A
small pre-B pool G
D
VH freq. in μMT non-productive VDJ
VH freq. in pro-B non-productive VDJ
Rsq = 0.76

 © The Author(s)

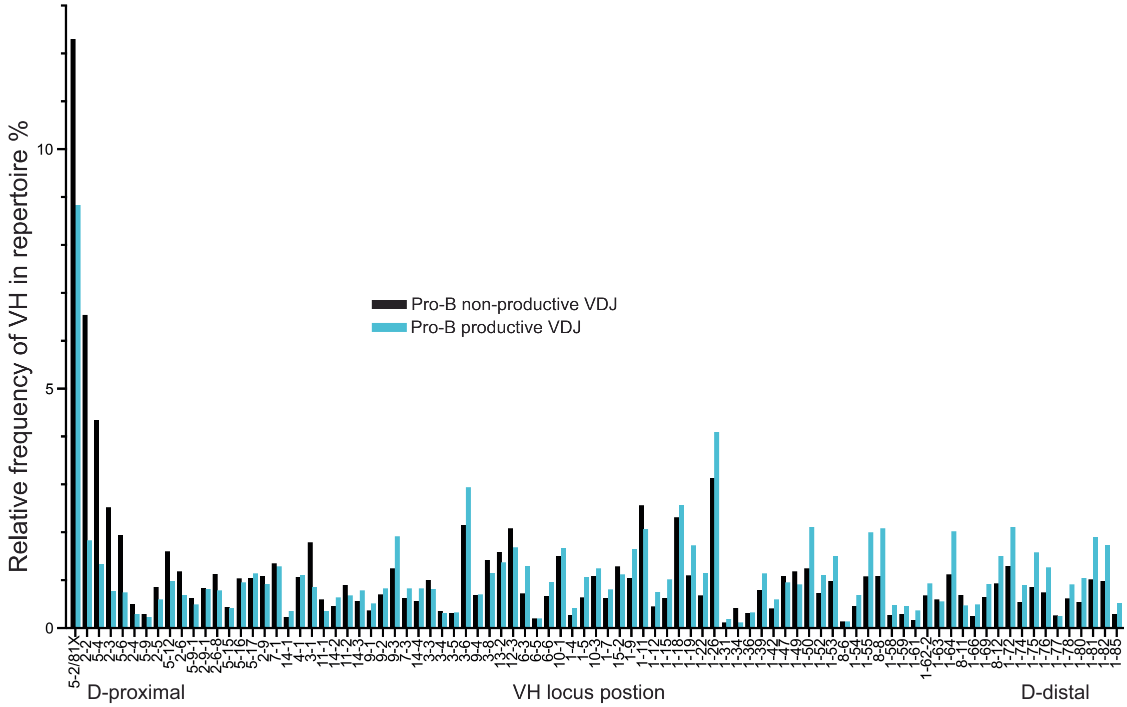

Figure EV2. VH selection in pro-B cells by locus position.

VH frequency in non-productive and productive VDJ from pro-B cells, by locus position, indicating the selection of productive VDJ that occurs prior to pre-BCR driven proliferation and differentiation into small pre-B cells. Data Information: Data derived from RStudio analysis of merged biological replicate datasets of VDJseq analysis of respective cell types, as Fig. 1. Source data are available online for this figure.

© The Author(s)

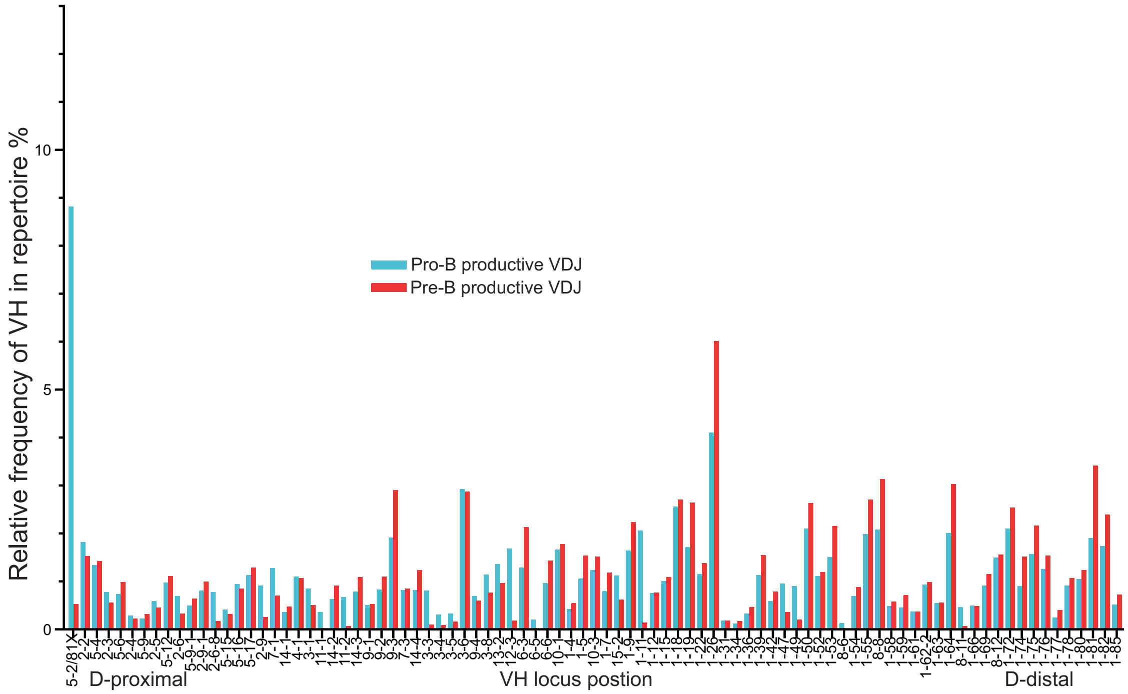

Figure EV3. VH selection after the pre-B transition.

VH frequency in productive VDJ from pro-B cells and pre-B cells, by locus position, indicating the further selection occurring to productive VDJ after completion of the pre-B transition. This selection is in addition to that shown in Figure EV2, together accounting for the overall selection shown in Fig. 2A. Data Information: Data derived from RStudio analysis of merged biological replicate datasets of VDJseq analysis of respective cell types, as Fig. 1. Source data are available online for this figure.

 © The Author(s)

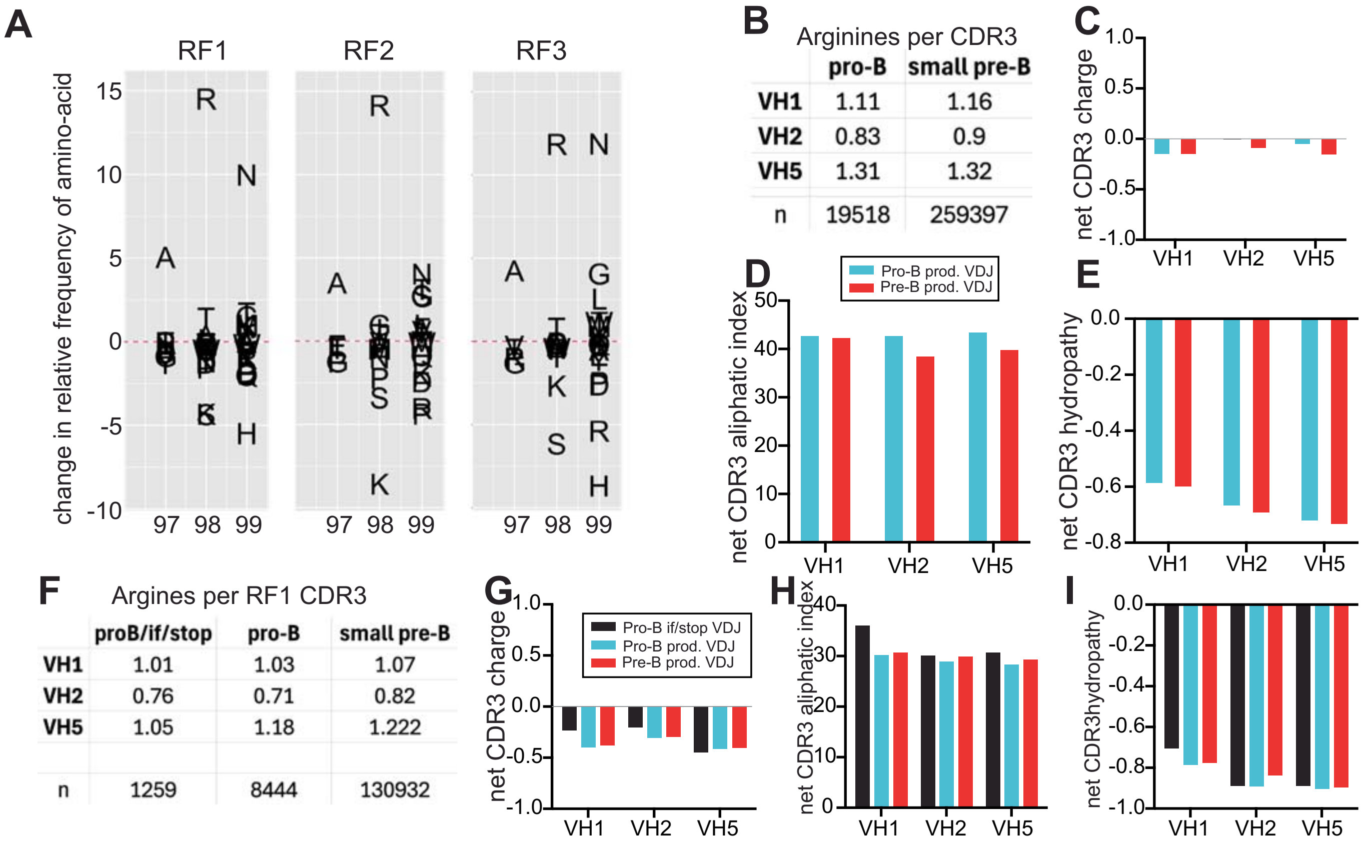

Figure EV4. Consistent selection of N-terminal CDR3 amino acids in all D reading frames, and biophysical properties of CDR3s, over the pre-B transition.

(**A**) Change in frequency of the first three CDR3 amino acid residues over the pre-B transition by D-reading-frame. Data shown here for VH2 only which also shows the strongest Y101 selection and RF2 counter-selection. (**B**) Mean number of Arginine residues per CDR3 for VH1, VH2, VH5 families from pro-B productive VDJ and pre-B productive VDJ. (**C**) Net CDR3 charge for VH1, VH2, VH5 families from pro-B productive VDJ and pre-B productive VDJ. (**D**) Net CDR3 aliphatic index for VH1, VH2, VH5 families from pro-B productive VDJ and pre-B productive VDJ. (**E**) Net CDR3 hydropathy for VH1, VH2, VH5 families from pro-B productive VDJ and pre-B productive VDJ. (**F**) Mean number of Arginine residues per RF1 CDR3 for VH1, VH2, VH5 families from pro-B non-productive (in-frame but with a stop codon), pro-B productive VDJ and pre-B productive VDJ. (**G**) Net RF1 CDR3 charge for VH1, VH2, VH5 families from pro-B non-productive (in-frame but with a stop codon), pro-B productive VDJ and pre-B productive VDJ. (**H**) Net RF1 CDR3 aliphatic index for VH1, VH2, VH5 families from pro-B non-productive (in-frame but with a stop codon), pro-B productive VDJ and pre-B productive VDJ. (**I**) Net RF1 CDR3 hydropathy for VH1, VH2, VH5 families from pro-B non-productive (in-frame but with a stop codon), pro-B productive VDJ and pre-B productive VDJ. Data Information: Panels A/B/F data derived from RStudio analysis of merged biological replicate datasets of VDJseq analysis of respective cell types, as Fig. 1. For panels C/D/E/G/H/I data was then further analysed using the method and algorithm from Protparam, https://web.expasy.org/protparam. Source data are available online for this figure.

© The Author(s)
